# Risk of injury to the femoral blood vessels based on the extent of acetabular dysplasia in total hip arthroplasty

**DOI:** 10.1007/s10047-019-01116-4

**Published:** 2019-07-05

**Authors:** Yuki Maeda, Nobuo Nakamura, Masaki Takao, Hidetoshi Hamada, Nobuhiko Sugano

**Affiliations:** 1 Department of Orthopaedic Medical Engineering, Osaka University Graduate School of Medicine, 2-2 Yamadaoka, Suita, Osaka 565-0871 Japan; 2 Center of Arthroplasty, Kyowakai Hospital, 1-24-1 Kishibekita, Suita, Osaka 564-0001 Japan; 3 Department of Orthopaedic Surgery, Osaka University Graduate School of Medicine, Suita, Osaka Japan

**Keywords:** Acetabular dysplasia, Injury to the femoral blood vessels, Total hip arthroplasty, Computed tomography-based navigation

## Abstract

We evaluated the course of the femoral blood vessels of patients with acetabular dysplasia. Patients were divided into five groups: those with Crowe type I, II, III, and IV dysplastic hips and those with normal hips. A computed tomography-based hip navigation software was used to measure the distance between the femoral blood vessels and the anterior pelvic wall in four axial planes located 10–40 mm proximal to the pelvic teardrop. In Crowe Groups I through IV, the distance was shortest at a point 20 mm proximal to the pelvic teardrop. Furthermore, the distance decreased as the Crowe classification grade increased. Because the femoral blood vessels pass close to the pelvis in many patients in Crowe III and IV hips, caution is required during surgery in these patients.

## Introduction

Total hip arthroplasty (THA) improves patients’ activities of daily living and can achieve high patient satisfaction [1]. As the number of older people increases worldwide, the number of THA cases is predicted to further increase by 2030 [2]. Although the incidence of injuries to the femoral blood vessels in THA reportedly ranges from 0.1 to 0.3% [3, 4], such injuries cause extremely serious complications.

We experienced a case of primary THA complicated with an intraoperative injury of the femoral artery. The patient had a Crowe type IV dysplastic hip and computed tomography (CT) images taken before the surgery showed that the femoral artery was closer to the acetabulum and more tortuous than normal. We thought that anterior capsular release by electrocautery may have injured the femoral artery due to the unusual closeness of the vessels to the capsule. This motivated us to evaluate the course of the femoral blood vessels based on the severity of acetabular dysplastic hips.

Fukunishi et al. [5] suggested that the course of the femoral blood vessels might be abnormal in the presence of abnormal bone morphology such as acetabular dysplasia. Bone morphology reportedly varies depending on the extent of acetabular dysplasia [6]. Therefore, we hypothesized that the anatomy of the femoral blood vessels may differ from that of the normal state in patients with severe acetabular dysplasia. To the best of our knowledge, very few reports have described the association between the extent of acetabular dysplasia and the anatomy of the blood vessels [5].

The purpose of this study was to evaluate the course of the femoral blood vessels based on the extent of acetabular dysplasia.

## Materials and methods

This retrospective cohort study was approved by our institutional review board. All patients included in the study had acquired preoperatively hip CT images for THA navigation at our hospital. All patients presented with severe hip osteoarthritis, and the severity of acetabular deformity was classified using the Crowe classification [7]. Because the numbers of Crowe Group III or IV patients were relatively smaller than those with Crowe Group I or II patients, our hip CT image database from 2006 to 2015 was used. The Crowe group I and II were created from the database by matching age and gender to the Crowe Group III and IV. As a control group, patients with a nondysplastic contralateral normal hip were chosen from among the patients with ipsilateral hip osteoarthritis in Crowe Groups I and II.

This selection process resulted in 108 patients for the analysis in this study. The patients comprised 9 men and 99 women with a mean age of 62.1 years (standard deviation 9.2 years; range 39–83 years), mean height of 154.8 ± 8.5 cm, mean weight of 57.0 ± 10.5 kg, and mean body mass index of 23.8 ± 3.5 kg/m^2^. The patients were classified into a control group (*n* = 20) and Crowe Groups I (*n* = 19), II (*n* = 24), III (*n* = 21), and IV (*n* = 24). There were no significant differences in sex, age, or body mass index among these five groups (unpaired *t* test) (Table 1).

**Table 1:** Patient characteristics in each group

Group (*n*)	Crowe Group I (*n* = 19)	Crowe Group II (*n* = 24)	Crowe Group III (*n* = 21)	Crowe Group IV (*n* = 24)	Control group (*n* = 20)
Male/female	3/16	2/22	0/21	4/20	0/20
Age (years)	62.6 ± 8.3	61.1 ± 10.0	58.5 ± 9.4	62.4 ± 9.5	65.2 ± 8.4
BMI (kg/m^2^)	23.4 ± 4.3	23.6 ± 3.6	24.6 ± 3.0	24.0 ± 3.6	23.5 ± 2.7

Data are presented as mean ± standard deviation

*BMI* body mass index

**Fig. 1:**
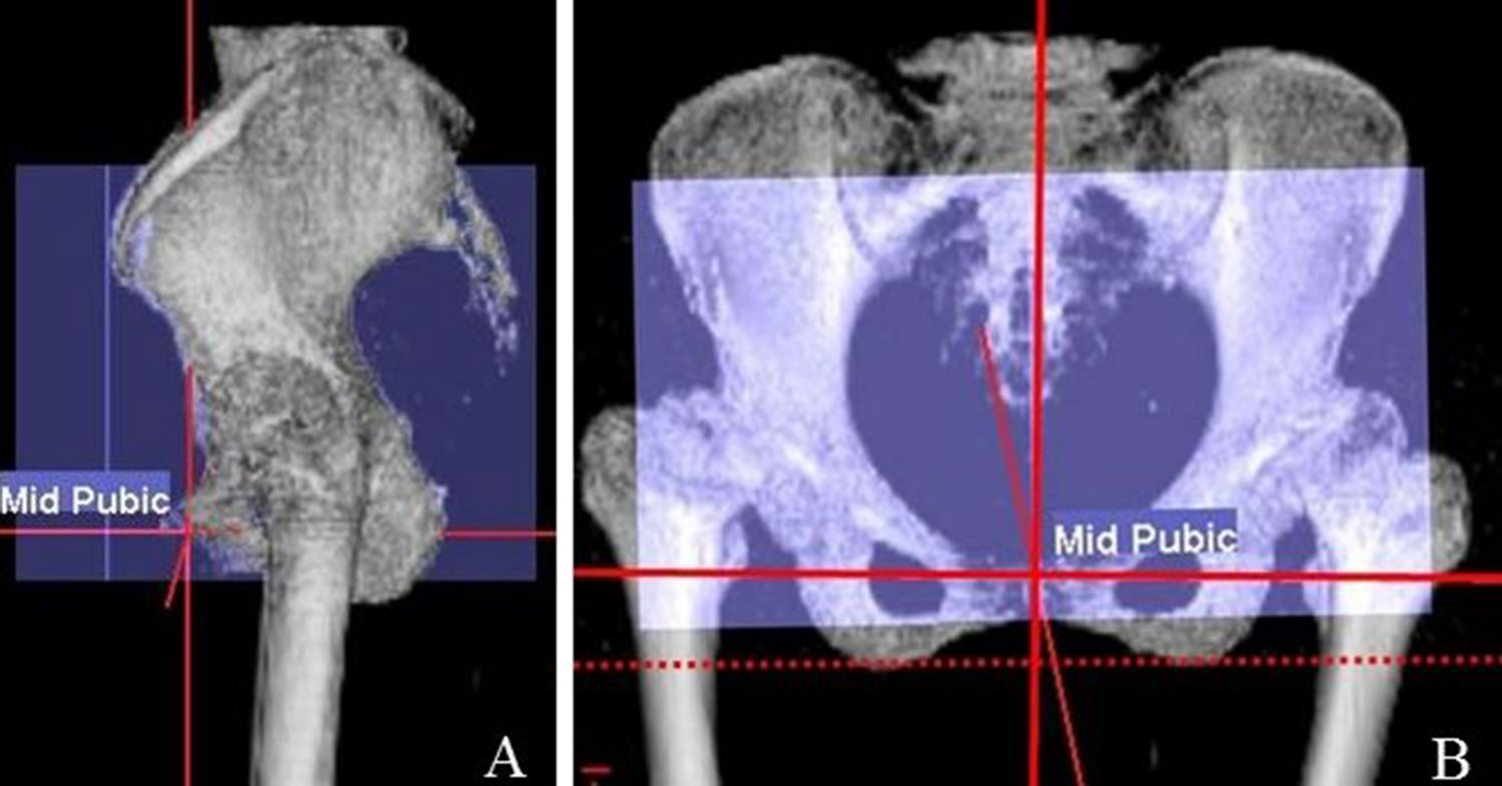
Patient positioning. **a** The pelvis was visualized in a functional plane in which the patients were placed in the supine position on the computed tomography (CT) scan table and the pelvis is axially rotated until the bilateral anterior superior iliac spines touch the same horizontal plane. **b** The pelvis was visualized in a functional plane, such that the lower edges of the bilateral ischia were horizontally aligned

**Fig. 2:**
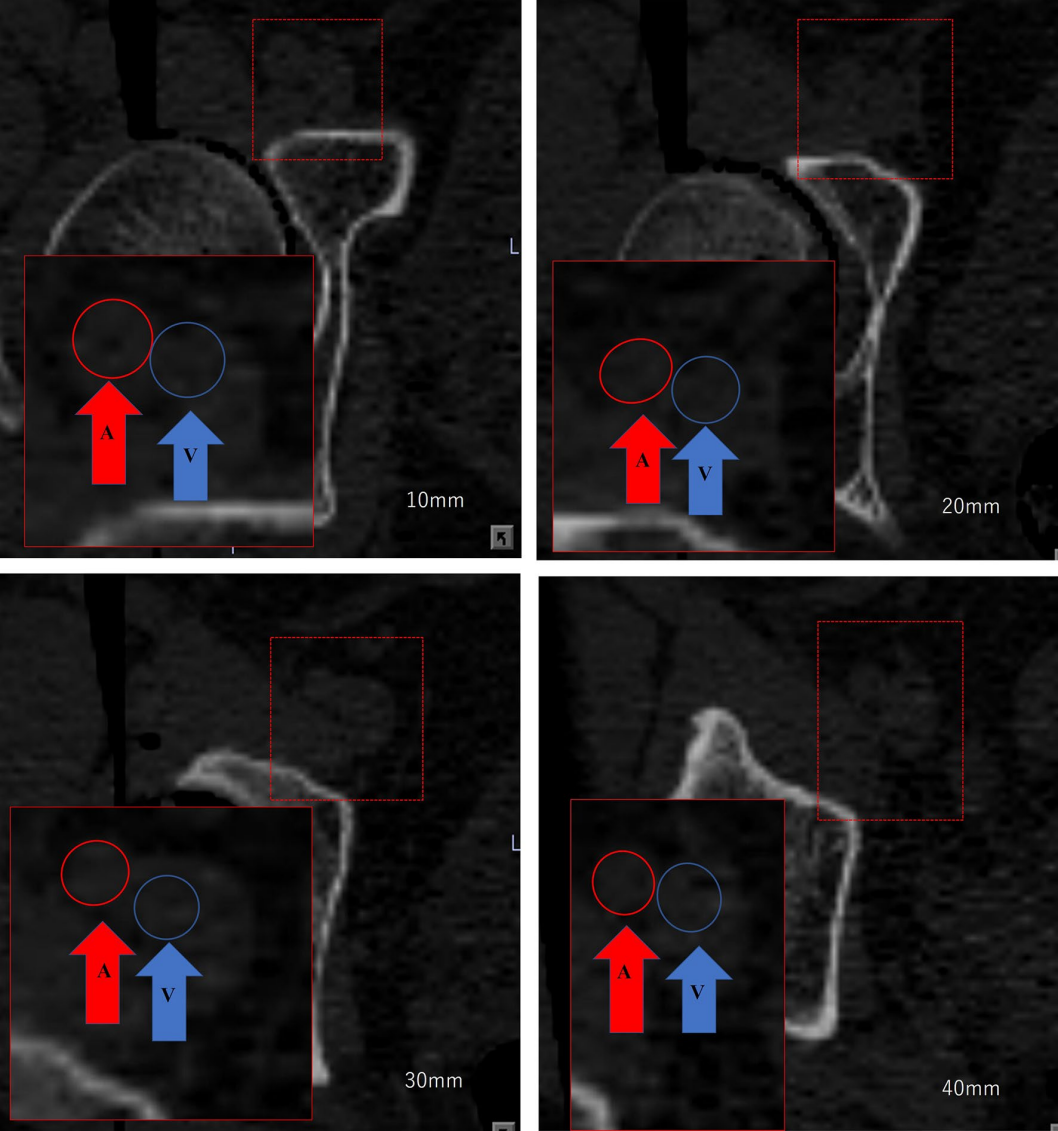
Four axial planes were created by positions 10–40 mm proximal to the teardrop. The distances to the femoral blood vessels at these points were used for analysis (red arrow shows the femoral artery and blue arrow shows the femoral vein. Red circle: femoral artery and blue circle: femoral vein)

Measurements were made on multiple planner reconstructed views of the pelvis using a CT-based navigation system software (CT Hip 1.1; Stryker, Mahwah, NJ). A functional coordinate of the pelvis was used to adjust the pelvic sagittal tilt in supine and the bilateral anterior superior iliac spines were used to determine the coronal plane (Fig. 1a). The lower edges of the bilateral ischia were used to define the horizontal axis (Fig. 1b) [8]. Based on the pelvic coordinates, four axial planes of the pelvis were reviewed at 10–40 mm proximal to the pelvic teardrop (Fig. 2 ). On these four axial planes, the distance between the femoral blood vessel and the anterior pelvic wall was measured. The femoral blood vessel was defined as follows. On the axial plane at the level of 10 mm proximal from the pelvic teardrop, the femoral blood vessels were easily identified as two circles located side by side under the skin (Fig. 2). Based on the initial identification of the femoral vessels, the femoral vessel contours were tracked serially on each axial plane above this level to confirm the femoral blood vessels on all planes.

**Fig. 3:**
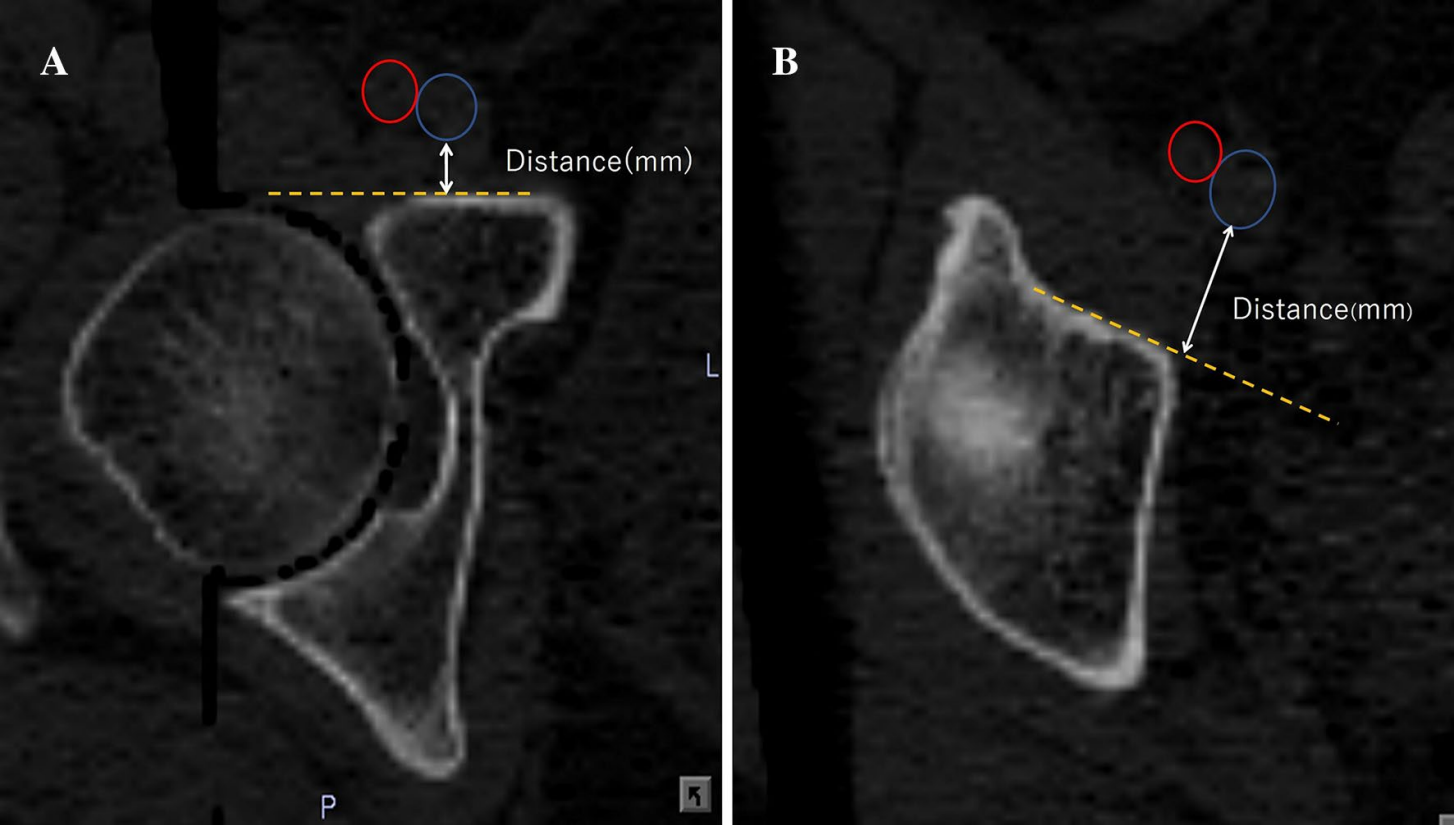
**a** Method of measurement of the distance between the anterior pelvic wall and the femoral blood vessels (red circle, femoral artery; blue circle, femoral vein). **b** An extension line (yellow dotted line) from the anterior pelvic wall was drawn when the anterior pelvic wall was small on axial planes that had a short distance from the pelvic teardrop

Similar to the method described by Fukunishi et al. [5], a perpendicular line was drawn from the femoral blood vessels to the anterior pelvic wall (Fig. 3a), and the shortest distance between the femoral blood vessels and the pelvis was measured. When the anterior pelvic wall was small on axial planes that had a short distance from the pelvic teardrop, an extension line from the anterior pelvic wall was drawn, and the distance between this line and the femoral blood vessels was measured (Fig. 3b). The distance between the femoral blood vessels and the pelvis was measured in the above-mentioned five groups.

All statistical analyses were performed with Ekuseru-Toukei 2012 (Social Survey Research Information Co., Ltd., Tokyo, Japan). Correlations among the groups were determined using the unpaired *t* test. Correlations were considered significant when the *p* value was < 0.05.

## Results

**Fig. 4:**
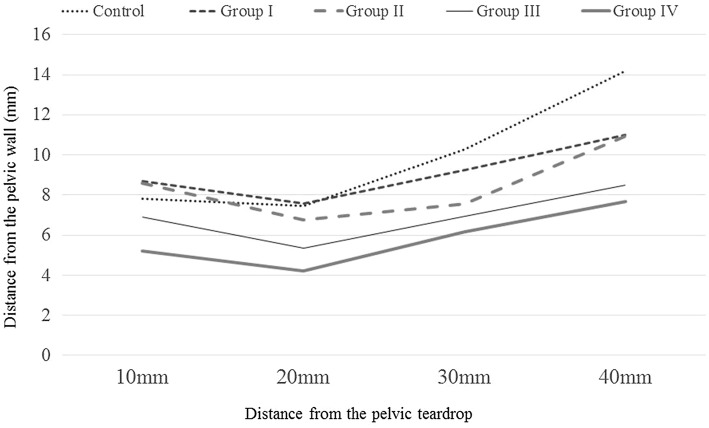
Distance between the femoral blood vessels and the anterior pelvic wall in Crowe Groups I–IV and the control group

The distance between the femoral blood vessels and the anterior pelvic wall in the five groups is shown in Fig. 4. In Crowe Groups I through IV, the distance between the femoral blood vessels and the anterior pelvic wall was shortest at a point 20 mm proximal to the pelvic teardrop. The mean distance in Crowe IV hips was 4.2 mm, that in Crowe I hips was 7.7 mm, and that in the control group was 7.5 mm. Furthermore, as the Crowe classification grade increased, the distance between the femoral blood vessels and the pelvis decreased.

The distances between the femoral blood vessels and the anterior pelvic wall are shown in Table 2. A clear and significant difference between the control group and Group I and between the control group and Group II was not observed in any of the axial planes. A significant difference was observed between the control group and Group III in all axial planes. Significant differences between the control group and Group IV were observed in all axial planes.

**Table 2:** Distance between femoral blood vessels and anterior pelvic wall

Group	Distance			
	10 mm	20 mm	30 mm	40 mm
Control group (mm)	7.8 ± 2.4	7.5 ± 2.2	10.3 ± 3.2	14.2 ± 2.9
Crowe Group I (mm)	8.9 ± 2.7	7.7 ± 3.0	9.5 ± 3.3	11.3 ± 3.6
Crowe Group II (mm)	8.6 ± 6.4	6.7 ± 2.6	7.6 ± 4.0	10.9 ± 3.6
Crowe Group III (mm)	6.9 ± 2.1*	5.4 ± 2.9*	6.9 ± 3.2*	8.5 ± 2.3*
Crowe Group IV (mm)	5.2 ± 1.9*	4.2 ± 1.7*	6.2 ± 2.7*	7.7 ± 3.5*

Data are presented as mean ± standard deviation

*p > 0.05 (unpaired *t* test) versus Control group

## Discussion

Vascular injuries during THA are extremely rare. Once they have occurred, however, the patient’s prognosis is poor, and other serious complications or sequelae may arise. Some case reports have described vascular injury during THA [3, 4, 9–11]. One reported cause of injury to the external iliac artery or femoral artery is inappropriate placement of the acetabular retractor [3, 10]. In one case, the femoral artery was injured due to the presence of a plaque during THA [11]. A systematic review of iatrogenic vascular injuries during THA indicated that vascular laceration was the most common cause of such injuries and that arterial injury accounted for > 80% of the cases [12]. Injury to the common femoral artery is observed most frequently, followed by the external iliac artery and the deep femoral artery. According to one review, although vascular injuries occur more commonly in revision cases, 50% of such injuries occur in primary cases [12].

Fukunishi et al. [5] performed three-dimensional CT angiography to visualize and assess the anatomy of the femoral blood vessels in patients who underwent complicated THA and required revision, those with severe dysplasia of Crowe Group III/IV, and those who had undergone arthrodesis. In their study, a distance of ≥ 10 mm to the anterior pelvic wall was obtained in one Crowe III hip and five Crowe IV hips. Although the authors assessed the femoral blood vessels using three-dimensional CT angiography, they evaluated only six cases of severely dysplastic hips. Furthermore, they did not describe how the pelvis was positioned in their study.

In the present study, the femoral blood vessels were clearly visualized even with plain CT up to the hip level. Moreover, the pelvis was visualized on a functional plane [13], such that the lower edges of the bilateral ischia were aligned horizontally. Every pelvis was evaluated in the same position, which was the strongest advantage of our analysis.

We showed that the distance to the anterior pelvic wall decreased at a point 20 mm proximal to the pelvic teardrop with a mean distance of < 5 mm, which is much shorter than that previously reported [5]. With the greater severity of dysplasia in the Crowe III or IV classification, the femoral blood vessels pass closer to the anterior pelvic wall. The mean distance between the femoral blood vessels and the pelvis was < 5 mm in the patients with Crowe IV hips. For these patients, careful attention is necessary when placing the acetabular retractor at the anterior pelvic wall. Moreover, performing the procedure too close to the anterior wall should be avoided when reaming near the original acetabulum or placing the cup.

We hypothesized that the cause of femoral blood vessels passing closer to the anterior wall is a severely acetabular dysplastic pelvis in which the femoral blood vessels are near the anterior wall of the original acetabulum; such patients can also present with anterior pelvic wall hypoplasia [6, 14] or iliopsoas atrophy caused by childhood disuse muscle atrophy.

A limitation of this study is that the femoral blood vessels may not have been appropriately assessed, because angiographic images were not taken. Nonetheless, the femoral blood vessels were clearly visualized even with plain CT up to the hip level. The second limitation is the small number of patients. This occurred, because fewer patients are classified as Crowe Group III or IV than as Crowe Group I or II, even in Japan. Finally, this was not a randomized-controlled study. However, the patients’ background characteristics, such as age, the ratio of males to females, and body mass index, were similar in this study.

## Conclusion

In conclusion, the femoral blood vessels pass close to the anterior pelvic wall in many patients with Crowe III and IV hips. Caution is, therefore, required during surgery in these patients.
